# Therapeutic comparison of a new budesonide/formoterol pMDI with budesonide pMDI and budesonide/formoterol DPI in asthma

**DOI:** 10.1111/j.1742-1241.2007.01574.x

**Published:** 2007-11

**Authors:** A H Morice, S Peterson, O Beckman, D Osmanliev

**Affiliations:** 1 Academic Medicine, University of Hull East Yorkshire, UK; 2 AstraZeneca R&D Lund, Sweden; 3 University Hospital of Pulmonary Diseases Sofia, Bulgaria

## Abstract

**Background:**

Budesonide/formoterol is an effective treatment for both asthma and chronic obstructive pulmonary disease. This study compared the efficacy and safety of a novel hydrofluoroalkane (HFA) pressurised metered-dose inhaler (pMDI) formulation of budesonide/formoterol with that of budesonide pMDI and budesonide/formoterol dry-powder inhaler (DPI; Turbuhaler®).

**Methods:**

This was a 12-week, multinational, randomised, double-blind, double-dummy study involving patients aged ≥ 12 years with asthma. All patients had a forced expiratory volume in 1 s of 50–90% predicted normal and were inadequately controlled on inhaled corticosteroids (500–1600 mu g/day) alone. Following a 2-week run-in, during which they received their usual medication, patients were randomised (two inhalations twice daily) to budesonide pMDI 200 mu g, budesonide/formoterol DPI 160/4.5 mu g or budesonide/formoterol pMDI 160/4.5 mu g. The primary efficacy end-point was change from baseline in morning peak expiratory flow (PEF).

**Results:**

In total, 680 patients were randomised, of whom 668 were included in the primary analysis. Therapeutically equivalent increases in morning PEF were observed with budesonide/formoterol pMDI (29.3 l/min) and budesonide/formoterol DPI (32.0 l/min) (95% confidence interval: −10.4 to 4.9; p = 0.48). The increase in morning PEF with budesonide/formoterol pMDI was significantly higher than with budesonide pMDI (+28.7 l/min; p < 0.001). Similar improvements with budesonide/formoterol pMDI vs. budesonide pMDI were seen for all secondary efficacy end-points. Both combination treatments were similarly well tolerated.

**Conclusions:**

Budesonide/formoterol, administered via the HFA pMDI or DPI, is an effective and well-tolerated treatment for adult and adolescent patients with asthma, with both devices being therapeutically equivalent.

**What's known**

The efficacy and safety of budesonide/formoterol administered via dry powder inhaler (DPI) are well established. However, pressurised metered-dose inhalers (pMDIs), the most commonly used inhalation devices in many countries worldwide, are currently being developed with hydrofluoroalkane (HFA)-based propellants to avoid the well-known ozone-depleting effects of chlorofluorocarbon propellants. Accordingly, budesonide/formoterol has also been reformulated as an HFA pMDI (Symbicort Rapihaler®).

**What's new**

The present study demonstrates that budesonide/formoterol administered via the novel HFA pMDI is an effective and well-tolerated treatment for adults and adolescents, and is therapeutically equivalent to budesonide/formoterol delivered via a DPI. These data provide further understanding of the use of the different budesonide/formoterol devices, and ultimately, will help provide clinicians and patients with greater freedom to select a delivery system that meets their needs and preferences.

## Background

Inhaled corticosteroid (ICS)/long-acting bgr _2_-agonist (LABA) therapies are the recommended maintenance treatment option for adults with persistent asthma (1). One such therapy is budesonide/formoterol in one dry-powder inhaler (DPI) (Symbicort® Turbuhaler®, AstraZeneca R&D, Lund, Sweden), a combination that has been shown in numerous trials to be an effective and well-tolerated treatment for asthma (2–7).

The pharmacological properties of budesonide/formoterol mean that this combination is suitable for use in different settings. The formoterol component is associated with a rapid onset of bronchodilatory effect, similar to that of standard, short-acting bgr _2_-agonist (SABA) reliever medications, such as salbutamol (8), as well as an extended duration of action, which is comparable with that of salmeterol (9). Budesonide, on the other hand, has a prolonged dwell time in the airway tissues, resulting in a long duration of anti-inflammatory effect (10–12). These properties mean that budesonide/formoterol is effective when given once daily (2) and when used in the acute setting (13). In addition, the nature of the dose–response curves of both budesonide and formoterol (14–16) means that temporarily increasing the dose of budesonide/formoterol in response to decreasing asthma control can provide patients with additional clinical benefits without increasing the risk of adverse systemic effects. As such, budesonide/formoterol is uniquely suitable for adjustable maintenance dosing (3, 4, 17, 18) and for use as maintenance and reliever therapy [Symbicort® Maintenance and Reliever Therapy (SMART)] (5–7, 19).

Although the use of DPIs, such as Turbuhaler®, is well established, pressurised metered-dose inhalers (pMDIs) are the most commonly used inhalation devices in many countries worldwide (20). The majority of available pMDI devices contain chlorofluorocarbon (CFC) propellants, which have well-documented adverse effects on the atmospheric ozone layer. Over the past few years, research has led to the development and approval of hydrofluoroalkane (HFA)-based aerosols – which do not have ozone-depleting properties – as alternatives to CFC propellants (21). Accordingly, budesonide/formoterol has also been reformulated as an HFA pMDI (Symbicort Rapihaler®) to provide clinicians and patients with greater freedom to select a delivery system that meets their needs and preferences.

The aim of this study was to compare the efficacy and safety of the new pMDI formulation of budesonide/formoterol with that of budesonide/formoterol DPI and budesonide (Pulmicort®, AstraZeneca R&D, Lund, Sweden) pMDI in adults and adolescents with asthma.

## Methods

### Patients

Adult and adolescent outpatients (aged ≥ 12 years) with asthma (22) for ≥ 6 months, who were inadequately controlled on ICS alone, were enrolled. For inclusion, patients had to have a forced expiratory volume in 1 s (FEV_1_) ≥ 50% and ≤ 90% of predicted normal (prebronchodilator), reversibility of ≥ 12% FEV_1_ after inhalation of terbutaline 1 mg (Bricanyl® Turbuhaler®, AstraZeneca) and a history of daily ICS use (stable dose of 500–1600 mu g/day within 30 days prior to enrolment) for ≥ 3 months.

Before randomisation, all patients had to have a total asthma symptom score ≥ 1 on ≥ 4 of the last 7 days of run-in (scale: 0 = no symptoms, 1 = aware of symptoms but can tolerate them easily, 2 = asthma causing enough discomfort to interfere with normal activities or sleep and 3 = unable to perform normal activities or sleep because of asthma; day- and nighttime scores summed). The first patient was enrolled on 30th April 2002 and the last patient completed the study on 6th February 2003.

### Study design

This was a 12-week, phase III, randomised, double-blind, double-dummy, parallel-group study conducted in 62 centres across eight countries (Brazil, Bulgaria, Canada, Hungary, Mexico, the Philippines, Thailand and the UK). The study complied with Good Clinical Practice guidelines and the ethical principles of the Declaration of Helsinki. An independent ethics committee or institutional review board approved the study protocol and patient consent form at each centre.

Patients were instructed to stop taking their LABA therapy for 3 days prior to the beginning of the run-in period (visit 1). Also, those using an ICS/LABA combination were requested to stop treatment with the combination 3 days before visit 1 and to continue with the same ICS alone. SABA use was restricted at 6 h prior to visit 1. During the study period [run-in and treatment period (visits 1–5)] any bgr _2_-agonists other than study reliever and reversibility test medication were not permitted.

Following a 10- to 14-day run-in, during which patients continued their prestudy ICS medication (stable dose), patients were randomised to treatment (two inhalations twice daily) with one of the following: budesonide pMDI 200 mu g (Pulmicort® pMDI); budesonide/formoterol DPI 160/4.5 mu g (Symbicort® Turbuhaler®); or budesonide/formoterol pMDI 160/4.5 mu g (Symbicort Rapihaler®). The doses of budesonide in each group were comparable; differences are explained by labelling changes for new inhaled drugs that require the delivered dose to be reported rather than the metered dose.

Patients were randomised sequentially in blocks of six using a computer-generated randomisation schedule. Eligible patients were consecutively allocated the lowest available randomisation code. The treatment code was only broken in the case of medical emergencies. The randomisation schedule was computer generated at AstraZeneca Research and Development, Charnwood, UK. To maintain blinding, each patient also received a placebo device. To reduce inconvenience, each patient received only two of the three devices: one active and one placebo device. An inhaled SABA (terbutaline 0.5 mg per inhalation or equivalent) was available for all patients for symptom relief.

### Assessments

The primary efficacy end-point was the change in morning peak expiratory flow (PEF) from baseline (mean of the last 10 days of run-in) to the mean value over the 12-week treatment period. Secondary efficacy end-points included: change from baseline (mean of the last 10 days of run-in) to the mean value over the treatment period in evening PEF; reliever medication use; reliever medication-free days; nighttime awakenings caused by asthma; asthma symptom score; symptom-free days (a night and day without asthma symptoms and no nighttime awakenings caused by asthma); and asthma-control days (a night and day without asthma symptoms or reliever medication use and no nighttime awakenings caused by asthma). All PEF measurements [taken prior to inhalation of study medication using a Mini-Wright® peak flow meter (Clement Clarke, Harlow, UK)], reliever medication use, nighttime awakenings caused by asthma and asthma symptom scores were recorded by patients in a daily diary.

Change from baseline (week 0) to the mean of the treatment period (weeks 2–12) in FEV_1_ and change from baseline (week 0) to the end of treatment (week 12) in Asthma Quality of Life Questionnaire (standardised version) [AQLQ(S)] scores (23) were also predefined secondary end-points. FEV_1_ was assessed during clinic visits at enrolment and randomisation and at 2, 6 and 12 weeks postrandomisation, according to European Respiratory Society recommendations (24, 25). The 32-item AQLQ(S) was administered during clinic visits at randomisation and at weeks 2 and 12 (seven-point scale: 1 = greatest possible impairment and 7 = least impairment) (23). A change in AQLQ(S) score of ≥ 0.5 units was defined as a clinically relevant change (26).

Safety assessments included adverse events (assessed throughout), vital signs (assessed at enrolment, randomisation, weeks 2 and 12) and clinical laboratory parameters (haematology, clinical chemistry and urinalysis; assessed at randomisation, weeks 2 and 12).

All patients received instruction on how to use the pMDI and DPI devices and the peak flow metre before the start of the study at visit 1. Each participating study site was provided with a Turbuhaler/pMDI to be used with disposable mouthpieces/actuators, allowing patients to practise the inhalation technique.

### Statistical analysis

The intent-to-treat (ITT) population (i.e. all randomised patients with postrandomisation data) was used for the main efficacy analyses. The primary objective of the study was to show that budesonide/formoterol pMDI was more efficacious than budesonide pMDI. The study was powered on this primary objective and approximately 600 evaluable patients (200 in each arm) were required for a 90% probability of detecting a true difference between budesonide/formoterol pMDI and budesonide pMDI of 13 l/min in mean change in morning PEF, assuming a standard deviation of 40 l/min (two-group *t*-test with a 5% two-sided significance level).

A secondary objective was to compare the efficacy of budesonide/formoterol pMDI with that of budesonide/formoterol DPI. Therapeutic equivalence between budesonide/formoterol pMDI and budesonide/formoterol DPI was considered to be established if the 95% confidence interval (CI) for the mean difference in morning PEF was within the prespecified equivalence limits of −15 and +15 l/min, as described previously (27). Assuming a standard deviation of 40 l/min, there was 90% probability of this CI being contained within these limits given that the actual difference was < 1.5 l/min. Results of the secondary analysis were not adjusted for multiplicity but a per-protocol (stability) analysis (excluding patients who violated the inclusion/exclusion or randomisation criteria) was performed to confirm the therapeutic equivalence data.

Diary-card end-points, averaged over available data, were analysed using a validated analysis of variance (ANOVA) model with treatment and country as fixed factors and the run-in mean (last 10 days) as a covariate. FEV_1_ and AQLQ(S) were also analysed using an ANOVA model with treatment and country as fixed factors and the randomisation value as a covariate. Safety variables were analysed using descriptive statistics.

## Results

### Patients

A total of 892 patients were enrolled and 680 were subsequently randomised to study treatment (217 to budesonide pMDI, 229 to budesonide/formoterol DPI and 234 to budesonide/formoterol pMDI). The ITT and safety populations comprised 679 patients (one patient in the budesonide/formoterol pMDI group was lost to follow-up). For the primary analysis, 216, 223 and 229 patients in the budesonide pMDI, budesonide/formoterol DPI and budesonide/formoterol pMDI groups, respectively, had morning PEF data from both the run-in and the treatment period. Six hundred patients completed the study; discontinuations were comparable between the three treatment groups (29, 23 and 27 for budesonide pMDI, budesonide/formoterol DPI and budesonide/formoterol pMDI, respectively).

Patients’ demographics and baseline characteristics were well balanced across the three treatment groups (Table 1). In total, 109 (16%) patients were aged 12–17 years, 520 (76%) were aged 18–64 years and 51 (8%) were aged ≥ 65 years; the distribution of adolescent and elderly patients was even across the three groups. Self-reported adherence to study medication (percentage of diary-logged days on which study medication was used) was equally high across all the three treatment groups (> 98%).

**Table 1:** Patient demographics and baseline characteristics

Characteristic	Budesonide pMDI (*n* = 217)	Budesonide/formoterol DPI (*n* = 229)	Budesonide/formoterol pMDI (*n* = 234)
Males/females, *n*	68/149	89/140	94/140
Mean age (range), years	40 (12–79)	39 (11–78)*	40 (12–78)
Smokers, *n* (%)	14 (6)	11 (5)	13 (6)
Median time since diagnosis (range), years†	10 (0–70)	9 (1–63)	8 (1–58)
Mean morning PEF (range), l/min	318 (109–638)	321 (93–668)	326 (89–715)
**Mean FEV**_1_**(range)**			
% predicted†	71 (45–91)	69 (50–90)	71 (39–92)
1	2.01 (0.85–4.25)	2.09 (1.05–3.75)	2.07 (0.94–4.12)
Mean ICS at entry (range), μg/day†	759 (400–1600)	774 (500–1600)	776 (400–1600)
LABA use at entry, *n* (%)	32 (15)	33 (14)	30 (13)
Reliever medication use (range), inhalations/day	2.0 (0.0–14.5)	1.8 (0.0–11.3)	2.1 (0.0–11.4)
Reliever medication-free days (range), %	29 (0–100)	34 (0–100)	29 (0–100)
Total asthma symptom score (range), 0–6	2.1 (0.4–5.7)	2.0 (0.0–6.0)	1.9 (0.0–5.3)
Nights with awakenings (range), %	33.1 (0–100)	32.1 (0–100)	29.2 (0–100)
Symptom-free days (range), %	10 (0–80)	12 (0–100)	12 (0–100)
Asthma-control days (range), %	8 (0–80)	10 (0–89)	10 (0–100)
AQLQ(S) (range), 1–7	4.80 (1.8–6.8)	4.62 (1.8–7.0)	4.70 (1.4–7.0)

* One patient was 11 years and 354 days old at the enrolment visit.

† Deviations from inclusion criteria not considered sufficiently significant to justify exclusion of data from the full analysis. AQLQ(S), Asthma Quality of Life Questionnaire (standardised version); DPI, dry-powder inhaler; FEV_1_, forced expiratory volume in 1 s; ICS, inhaled corticosteroid; LABA, long-acting β_2_-agonist; PEF, peak expiratory flow; pMDI, pressurised metered-dose inhaler.

### Efficacy

**Figure 1:**
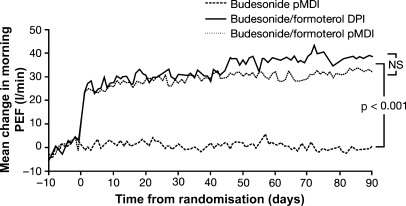
Change in morning PEF following treatment with budesonide pMDI, budesonide/formoterol DPI or budesonide/formoterol pMDI. DPI, dry-powder inhaler; NS, not significant; PEF, peak expiratory flow; pMDI, pressurised metered-dose inhaler

Budesonide/formoterol DPI and budesonide/formoterol pMDI improved morning PEF compared with budesonide pMDI (p < 0.001 for both) (Figure 1). Following treatment, the adjusted mean change in morning PEF was 31.4 and 28.6 l/min higher in the budesonide/formoterol DPI and budesonide/formoterol pMDI groups, respectively, than in the budesonide pMDI group. Analysis of improvements from baseline in morning PEF with budesonide/formoterol DPI and budesonide/formoterol pMDI established that the two treatments were therapeutically equivalent (Table 2). A stability analysis of improvements from baseline in morning PEF in the per-protocol population confirmed the therapeutic equivalence of the two budesonide/formoterol inhalation devices; the adjusted mean difference between budesonide/formoterol pMDI and budesonide/formoterol DPI was −4.9 l/min (95% CI: −12.8 to 3.0; p = 0.22).

**Table 2:** Therapeutic equivalence of budesonide/formoterol DPI and budesonide/formoterol pMDI

	Morning PEF (l/min)		
	
Comparison	Adjusted mean difference	95% CI	p-value
Budesonide/formoterol pMDI vs. budesonide/formoterol DPI	−2.8	−10.4 to 4.9*	0.48
Budesonide/formoterol pMDI vs. budesonide pMDI	28.6	20.9–36.4	< 0.001
Budesonide/formoterol DPI vs. budesonide pMDI	31.4	23.7–39.2	< 0.001

* Therapeutic equivalence was defined as a 95% CI for the difference in morning PEF between budesonide/formoterol pMDI and budesonide/formoterol DPI within the range −15 to +15 l/min. CI, confidence interval; DPI, dry-powder inhaler; PEF, peak expiratory flow; pMDI, pressurised metered-dose inhaler.

**Figure 2:**
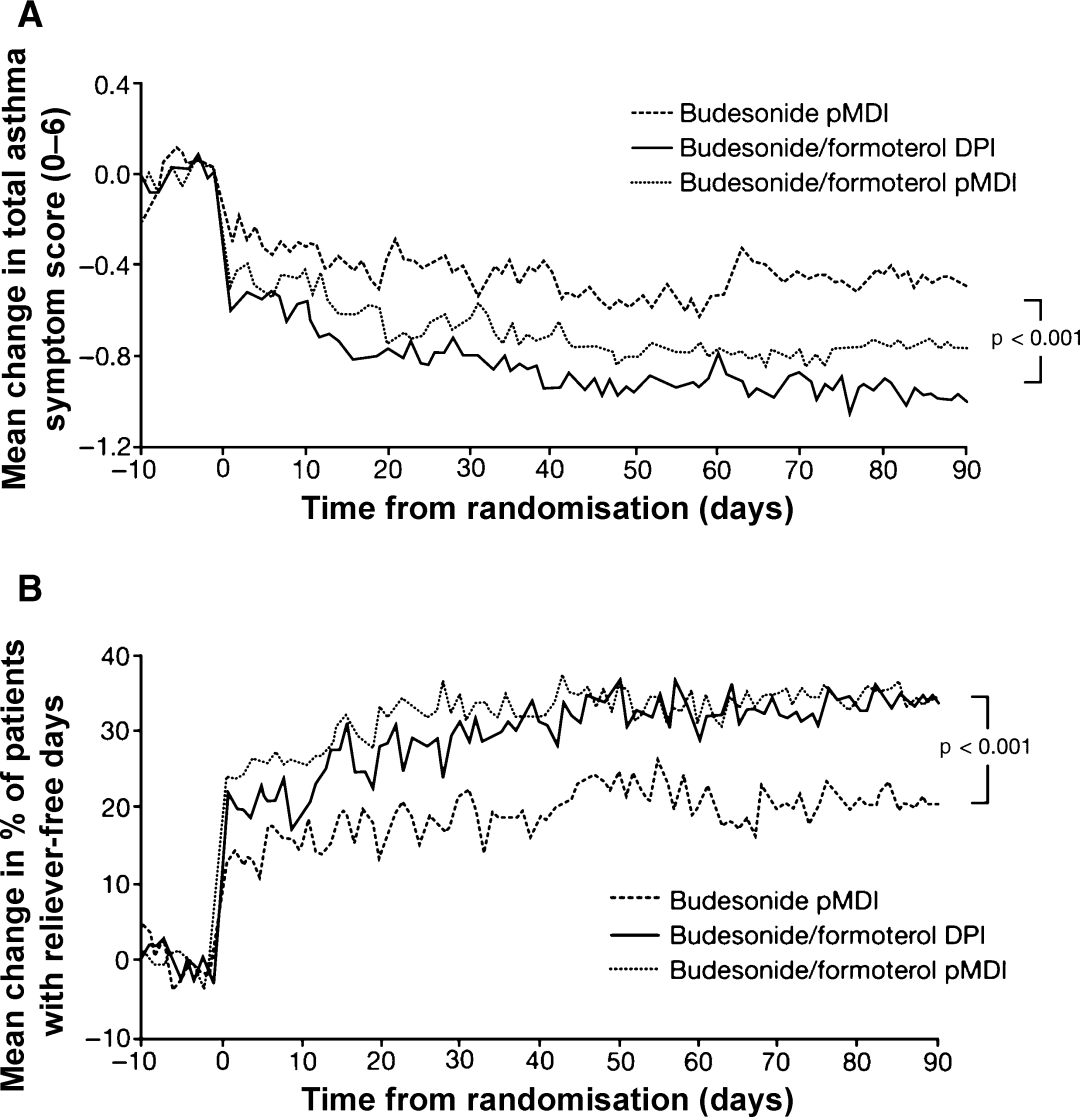
Change in (A) total asthma symptoms and (B) reliever medication-free days following treatment with budesonide pMDI, budesonide/formoterol DPI or budesonide/formoterol pMDI. DPI, dry-powder inhaler; pMDI, pressurised metered-dose inhaler

**Figure 3:**
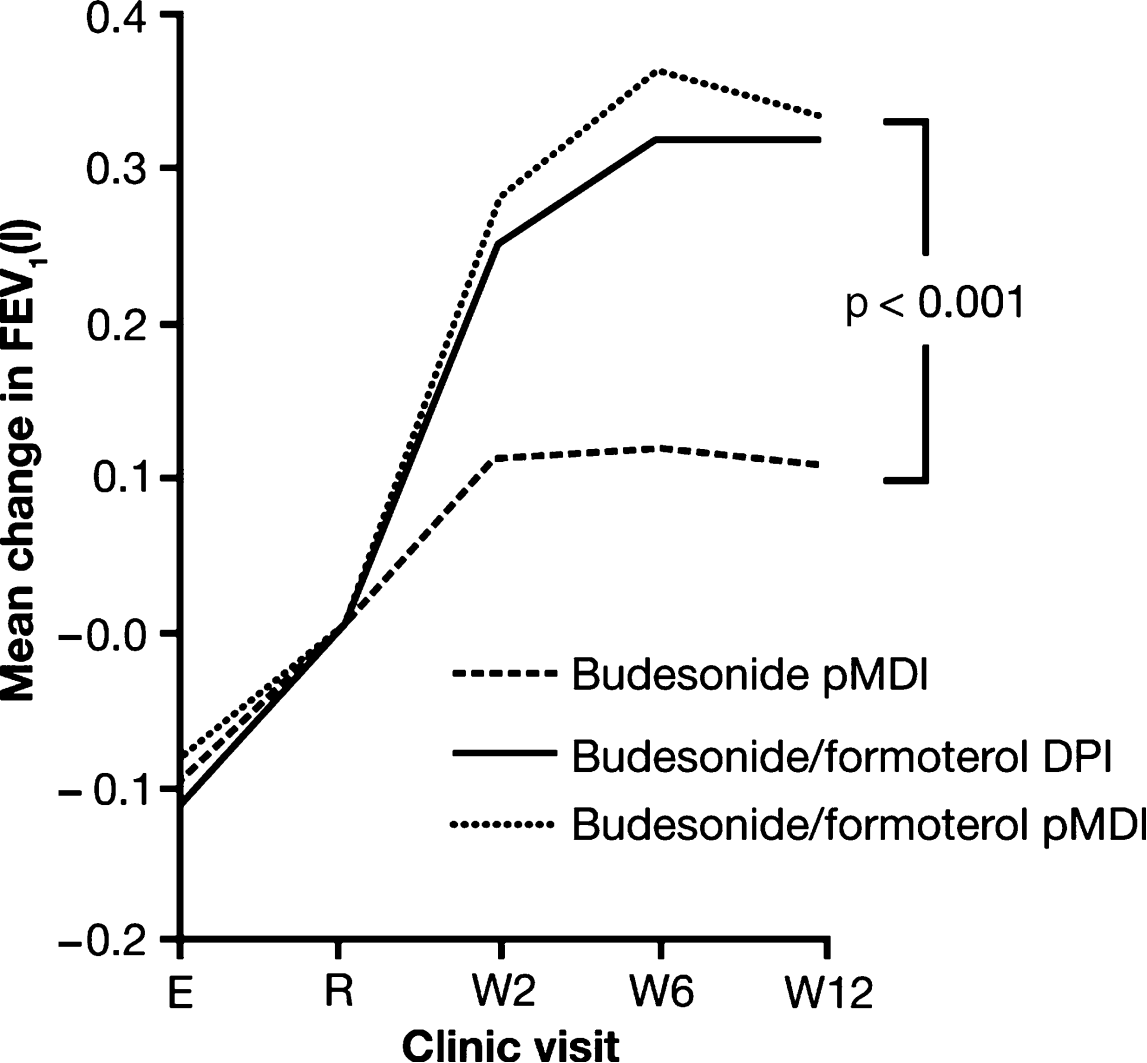
Change in FEV_1_ following treatment with budesonide pMDI, budesonide/formoterol DPI or budesonide/formoterol pMDI. DPI, dry-powder inhaler; E, enrolment; FEV_1_, forced expiratory volume in 1 s; pMDI, pressurised metered-dose inhaler; R, randomisation; W, week

All secondary diary end-points were improved to a greater extent with budesonide/formoterol DPI and budesonide/formoterol pMDI than with budesonide pMDI (Table 3; Figure 2). For most end-points, the improvements seen with budesonide/formoterol pMDI were similar to those observed with budesonide/formoterol DPI, with the exception of symptom-free and asthma-control days, which were increased by a slightly greater degree with budesonide/formoterol DPI (Table 3). Consistent with improvements in diary end-points, both budesonide/formoterol DPI and budesonide/formoterol pMDI also improved FEV_1_ compared with budesonide pMDI (Figure 3), with no significant difference between the two budesonide/formoterol devices.

**Table 3:** Changes in secondary diary end-points following treatment with budesonide pMDI, budesonide/formoterol DPI or budesonide/formoterol pMDI

	Adjusted mean change from run-in		
	
Variable	Budesonide pMDI (*n* = 217)	Budesonide/formoterol DPI (*n* = 229)	Budesonide/formoterol pMDI (*n* = 233)
Evening PEF, l/min	−0.6	25.1*	24.3*
Reliever medication use, no. of inhalations/24 h	−0.35	−0.92*	−0.94*
Reliever medication-free days, %	17.9	31.1*	30.8*
Total asthma symptom score, 0–6	−0.44	−0.84*	−0.70*
Nights with awakenings, %	−9.7	−15.5†	−16.5*
Symptom-free days, %	19.1	34.2*††	28.0†
Asthma-control days, %	18.3	33.1*††	26.5†

* p < 0.001

† p < 0.01 vs. budesonide pMDI

†† p < 0.05 budesonide/formoterol DPI vs. budesonide/formoterol pMDI. DPI, dry-powder inhaler; PEF, peak expiratory flow; pMDI, pressurised metered-dose inhaler.

### Health-related quality of life

**Figure 4:**
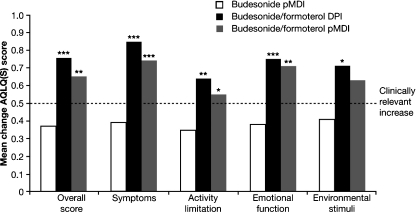
Change in AQLQ(S) following treatment with budesonide pMDI, budesonide/formoterol DPI or budesonide/formoterol pMDI. AQLQ(S), Asthma Quality of Life Questionnaire (standardised version); DPI, dry-powder inhaler; pMDI, pressurised metered-dose inhaler. ***p < 0.001, **p < 0.01, *p < 0.05 vs. budesonide pMDI

Budesonide/formoterol DPI and budesonide/formoterol pMDI provided similar clinically relevant improvements in health-related quality of life (Figure 4). These improvements with budesonide/formoterol DPI and budesonide/formoterol pMDI were statistically greater than those provided by budesonide pMDI [adjusted mean change in AQLQ(S) overall score: +0.76 (p < 0.001 vs. budesonide pMDI), +0.65 (p = 0.002 vs. budesonide pMDI) and +0.37, respectively]. For the overall AQLQ(S) score, 52% and 56% of budesonide/formoterol pMDI-treated and budesonide/formoterol DPI-treated patients, respectively, had a clinically relevant increase of ≥ 0.5 units compared with 35% of patients in the budesonide pMDI group.

### Safety

There were no clinically important differences between treatment groups with regard to adverse events [overall (Table 4) and ICS- and bgr _2_-agonist-related events (Table 5)], vital signs or laboratory parameters. Only 32% of patients experienced one or more adverse events, the majority of which were mild or moderate in severity. Four patients reported serious adverse events: two in the budesonide pMDI group (joint dislocation/accident/fracture, asthma aggravated) and two in the budesonide/formoterol pMDI group (menorrhagia, increase in liver enzyme activity); none were considered (after follow-up) to be related to study treatment. No deaths were reported.

**Table 4:** Most frequently reported adverse events

	No. of patients (%)*		
	
Adverse event	Budesonide pMDI (*n* = 217)	Budesonide/formoterol DPI (*n* = 229)	Budesonide/formoterol pMDI (*n* = 233)
Patients with ≥ 1 event	82 (38)	66 (29)	70 (30)
Nasopharyngitis	17 (8)	6 (3)	4 (2)
Upper respiratory tract infection	9 (4)	9 (4)	7 (3)
Pharyngitis	7 (3)	8 (3)	4 (2)
Lower respiratory tract infection	6 (3)	4 (2)	2 (1)
Headache	4 (2)	4 (2)	4 (2)
Influenza	5 (2)	2 (1)	4 (2)
Wheezing	5 (2)	3 (1)	2 (1)
Oral candidiasis	3 (1)	5 (2)	2 (1)
Cough	5 (2)	2 (1)	3 (1)
Asthma aggravated	7 (3)	2 (1)	1 (< 0.5)

* Only adverse events reported for ≥ 10 patients in total are included. DPI, dry-powder inhaler; pMDI, pressurised metered-dose inhaler.

**Table 5:** Incidence of adverse events related to treatment with bgr _2_-agonists or ICS

	No. of patients (%)		
	
Adverse event	Budesonide pMDI (*n* = 217)	Budesonide/formoterol DPI (*n* = 229)	Budesonide/formoterol pMDI (*n* = 233)
**ICS-related adverse events**			
Dysphonia	1 (< 0.5)	2 (1)	1 (< 0.5)
Oral candidiasis	3 (1)	5 (2)	2 (1)
β_2_**-agonist-related adverse events**			
Tremor	1 (< 0.5)	2 (1)	0
Palpitation	0	6 (3)	1 (< 0.5)
Headache	4 (2)	4 (2)	4 (2)

DPI, dry-powder inhaler; ICS, inhaled corticosteroid; pMDI, pressurised metered-dose inhaler.

Thirty patients (15 in the budesonide pMDI group, four in the budesonide/formoterol DPI group and 11 in the budesonide/formoterol pMDI group) discontinued the study because of adverse events. The most frequently reported adverse event causing discontinuation was asthma aggravated [seven, two and one patient(s) in the budesonide pMDI, budesonide/formoterol DPI and budesonide/formoterol pMDI groups, respectively]. Other adverse events leading to discontinuation included nausea, tremor, palpitations and lower respiratory tract infection.

## Discussion

Budesonide/formoterol, which is available as a DPI, has been reformulated as an HFA pMDI to enable delivery of this effective and well-tolerated therapy via two different devices that meet the needs and requirements of both patients and clinicians alike without detriment to the environment. This large-scale, international, double-blind, double-dummy study set out to compare the efficacy and safety of this novel pMDI formulation of budesonide/formoterol with that of the established DPI, budesonide/formoterol Turbuhaler®, and budesonide pMDI in adults and adolescents with inadequately controlled asthma.

Analysis of the primary end-point – morning PEF – demonstrated that both budesonide/formoterol therapies are more effective than budesonide pMDI. The magnitude of improvement in morning PEF with both budesonide/formoterol therapies was within the range of that reported previously for budesonide/formoterol DPI (2, 17, 28, 29), thus verifying the results of the present study. Furthermore, the analysis of the secondary objective demonstrated that, for improvements in morning PEF, budesonide/formoterol pMDI is therapeutically equivalent to budesonide/formoterol DPI.

Analysis of other efficacy end-points relating to lung function, asthma symptoms, disease control and health-related quality of life supports the comparable efficacy of budesonide/formoterol pMDI with budesonide/formoterol DPI and the superiority of both formulations over budesonide pMDI. The only statistical differences between the two devices – which favoured budesonide/formoterol DPI – were in the composite end-points: symptom-free days and asthma-control days. In both cases, differences were driven by the additional improvement in daytime total asthma symptom score (data not shown). Previous studies report that the degree of lung deposition for the DPI device is approximately 2–3 times that of the corresponding pMDI device (30–32) and hence it is possible that this may have contributed to the significant differences in symptom-free days and asthma-control days observed in this study. However, there is no existing literature regarding the relative degree of lung deposition for budesonide/formoterol DPI or pMDI and as the degree of lung deposition varies for different DPIs and pMDIs it cannot be assumed that Turbuhaler DPI will deliver 2–3 times more drug to the lungs when compared with a pMDI (33). Instead it is more likely that any improvements in asthma symptoms associated with DPI are due to random variation and a much larger study would be required to detect a clear difference in outcome for these variables. Furthermore, as there were no differences between the two devices in end-points such as nighttime awakenings, 24-h total asthma symptom scores and reliever medication use (markers of asthma control), and in the number of adverse events reported as asthma aggravated (an indicator of more severe events), it is unlikely that the difference in daytime symptom variables is clinically important. Interestingly, the improvements in lung function and symptom-related end-points with both budesonide/formoterol therapies appeared to be progressive, with the evidence of continuing improvement between weeks 2 and 6 (Figures 1–3). Conversely, but perhaps not unexpectedly, there appeared to be little evidence of progressive improvement with budesonide pMDI in this patient population. As previously reported in comparative studies of budesonide/formoterol DPI vs. budesonide DPI (2, 18, 34, 35), and as would be expected for an ICS (1), budesonide pMDI improved asthma symptoms, measures of asthma control and health-related quality of life, but these improvements were smaller than those seen with the budesonide/formoterol therapies.

In the clinical setting, choice of inhaler device is less likely to depend on efficacy and tolerability and is more likely to be influenced by other factors, for example cost, the patient's ability to use the selected device correctly and personal preference (36). DPI devices are generally more expensive than pMDIs and the therapeutic equivalence observed between the delivery devices suggests that either would be suitable for use in normal clinical practice. Besides cost, differences in ease of use and technique between pMDI and the DPI are likely to have a large influence on choice of inhaler for individual patients and clinicians. Although both devices are relatively easy to use, each has technical limitations which can limit effectiveness (37). In this study patients were instructed on how to use the inhalers correctly at visit 1. Each participating study site was provided with a DPI/pMDI to be used with disposable mouthpieces/actuators, allowing patients to practise the inhalation technique. Furthermore, results report that compliance (study drug use measured by self-reported diary recording of daily treatment intake) was similar across all treatment groups. Thus, it is unlikely that patients randomised to receive the pMDI inhaler had any disadvantages compared with those using the DPI with regard to inhaler technique. In the clinical setting, providing each individual with the most appropriate inhaler has the potential to result in more effective patient care (37).

In terms of safety, budesonide/formoterol pMDI, budesonide/formoterol DPI and budesonide pMDI were well tolerated, with a low overall incidence of adverse events across the three treatment groups. Adverse events were slightly more common in the budesonide pMDI group – although this was not thought to be clinically relevant – and the majority of patients who reported aggravated asthma as an adverse event were in this treatment group. Importantly, the tolerability profiles of both budesonide/formoterol therapies were similar to that reported previously for budesonide/formoterol DPI (29).

In conclusion, this study demonstrates that budesonide/formoterol, administered via the HFA pMDI or DPI, is an effective and well-tolerated treatment for adult and adolescent patients with asthma, with both devices being therapeutically equivalent. The availability of both devices will give clinicians greater freedom to select a cost-effective therapy that suits the needs and preferences of individual patients and clinicians themselves.
